# “The Idea of Being Without It is Frightening”. Uncertainty and Psychological Dependency for Patients Using Immuno‐, Biological, or Precision Therapies: A Qualitative Study

**DOI:** 10.1002/pon.70318

**Published:** 2025-11-08

**Authors:** J. Lecouturier, L. Crowe, M. C. Brown, A. Greystoke, A. Bojke, R. Bojke, J. Richardson, M. Wells, E. Ezeala, L. Carter, L. Sharp, A. Todd

**Affiliations:** ^1^ Population Health Sciences Institute Newcastle University Newcastle Upon Tyne UK; ^2^ Newcastle University Centre for Cancer Newcastle University Newcastle Upon Tyne UK; ^3^ Northern Centre for Cancer Care Freeman Hospital Newcastle Upon Tyne Hospitals NHS Foundation Trust Newcastle Upon Tyne UK; ^4^ Patient and Public Involvement Newcastle Upon Tyne UK; ^5^ Nursing Directorate Imperial College Healthcare NHS Trust London UK; ^6^ Department of Surgery and Cancer Imperial College London London UK; ^7^ Department of Pharmacy North Tyneside General Hospital Northumbria Healthcare NHS Foundation Trust North Shields UK; ^8^ Division of Cancer Sciences Faculty of Biology Medicine and Health The University of Manchester Manchester UK; ^9^ The Christie NHS Foundation Trust Manchester UK; ^10^ School of Pharmacy Newcastle University Newcastle Upon Tyne UK

## Abstract

**Background:**

IBP therapies (immunotherapies, biologics and precision therapies) have had a significant impact on cancer survival. They differ from conventional cancer treatments in that they may be used long term and have less predictable adverse effects. We aimed to explore the psychological impact of IBP therapies from the perspectives of those living with advanced cancer.

**Methods:**

Semi‐structured interviews with 20 women and 11 men with advanced cancer ‐ (lung, colorectal, ovary, (female) breast, renal and malignant melanoma) ‐ about their experiences of IBP therapies. Data were analysed thematically.

**Results:**

We elicited two overarching themes from the data: (1) the psychological impact of a stage IV diagnosis and living with advanced cancer, and (2) uncertainty and dependency associated with IBP therapies. Although some were relieved to have a diagnosis, others responded with shock and disbelief. Day to day, cancer was foremost in the minds of many, and the remainder chose to push these thoughts aside and focus on making the most of life. Living with cancer led to a shift in their sense of self. Uncertainty and dependency throughout the care pathway were centred around treatment effectiveness, heightened by the belief that the therapy was keeping them alive. Participants were uncertain and anxious about any interruption of, or changes to, their current treatment regimen, stopping their current treatment and next steps.

**Conclusion:**

Interventions are needed to support the psychological needs of people using IBP therapies. These should go beyond monitoring and managing IBP treatment‐related adverse effects and address uncertainty and psychological dependency.

## 1 Background

In recent years, there have been important advancements in the pharmacological approaches to treat many different cancers, including the immunotherapies (which stimulate or restore the ability of the immune system to detect and destroy cancer cells), the biologics (monoclonal antibodies that block key pathways for cancer growth or survival) and the precision therapies (tailoring a treatment to account for genomic differences of the cancer) [1, 2]. These new treatment approaches, collectively known as the immuno‐, biological or precision (IBP) therapies, have transformed the cancer treatment landscape; people with advanced cancer can often survive for years, instead of months [3]. For more detailed information about the IBP therapies, please see reviews by Zhang et al., [4] and Min and Lee [5].

In comparison to conventional cytotoxic cancer treatments, there are some important differences in how IBP therapies are used. For example, some IBP therapies are used long‐term, whereas cytotoxic cancer treatments are used on a cyclical basis, typically over a period of weeks or months. Another difference is the adverse effect profile: cytotoxic treatments have well characterised adverse effects, including nausea, vomiting, neutropenia, and hair loss [2], while the IBP therapies have wider ranging, variable and sometimes less predictable adverse effects [6, 7, 8]. This changing treatment paradigm may have important implications for how people experience their cancer treatment(s).

Literature exploring the patient experience of using IBP therapies has started to emerge. A qualitative systematic review exploring patient experiences of using checkpoint inhibitors (a type of immunotherapy) identified themes related to treatment decision‐making, success of treatment, adverse effects, and quality‐of‐life, and concluded there remains scope for further qualitative work in this area [9]. One area that remains underexplored is how the changing cancer treatment landscape—from conventional cytotoxic treatment to IBP therapy—impacts on the psychological challenges for people living with advanced cancer. As part of a larger project, the TARGET study [10, 11], this work aimed to address this knowledge gap by exploring the psychological impact of using IBP therapy from the perspectives of people living with advanced cancer.

## 2 Methods

### 2.1 Design

This was a qualitative study, using in‐depth semi‐structured interviews. The target population was adults diagnosed with advanced cancer treated with an IBP therapy, for at least 1 month. For maximum variation, a purposeful sampling strategy was used, with the primary sampling strata being cancer type and sex. Cancer site included colorectal, ovary, (female) breast, renal and malignant melanoma, as the treatment regimens often include IBP therapies. Participants could have previously undergone other cancer treatments (e.g., surgery, chemotherapy). However, a period of at least 3 months had to have passed to help focus the interviews on IBP therapies rather than earlier treatments.

Between June 2021 and February 2022 participants were recruited from one of two routes: (1) through research support staff or cancer health care professionals from four National Health Service (NHS) Hospital Foundation Trusts in England who screened patient records and provided eligible patients with study information; and (2) through cancer charities where those interested were asked to contact the research team and were then screened for eligibility. Three of the hospital sites were located in cities and one in a small town, and the catchment areas of the hospitals encompassed a mix of urban areas, towns, villages and rural areas. The Tyne and Wear South Research Ethics Committee (REC reference: 21/NE/0028) gave a favourable ethical opinion.

### 2.2 Data Collection

A topic guide was developed and reviewed by patient and public collaborators and discussed at a regional cancer support group. Four main areas were included in the topic guide: receiving a cancer diagnosis; understanding of treatment; impact of treatment/cancer; and any support sought and/or received. The guide was used flexibly, to enable participants to raise topics important to them. As data collection progressed, any novel issues raised were added to the topic guide and explored in subsequent interviews. Depending on patient preference, interviews were conducted face to face in the participant's home, by telephone or a video conference platform, by two female researchers (LC and MCB) experienced in qualitative research methods, unknown to participants. Informed consent was obtained, and interviews were digitally audio recorded with the permission of the participant. As a token of appreciation, interview participants were offered a £20 gift voucher. Interview duration ranged from 24 to 122 min (average 71 min). Sound files were transcribed verbatim, anonymised and uploaded to NVivo which was used as a data management tool.

Interviews continued until data saturation was achieved and no new themes were identified.

### 2.3 Analysis

Data were analysed thematically [12]. A framework was developed from a small number of interview transcripts; this was tested against another batch and the framework amended accordingly. The full dataset was coded using this framework (JL). Emergent themes and sub‐themes were discussed and agreed with the wider team.

## 3 Findings

Thirty‐one individuals (20 female, 11 male) were interviewed (Table 1). Participants described their cancer journey from diagnosis to the present day including their experience of IBP therapies. Although reports of the impact of the diagnosis and living with advanced cancer were similar to earlier findings [13, 14, 15, 16, 17], a brief description is included to provide context. The main focus of this work is living with uncertainty and dependency associated with the IBP therapies.

**TABLE 1:** Participant characteristics.

Sex	
Female	20
Male	11
Recruitment route	
NHS Hospital	27
Cancer charity	4
Age range	
31–40	1
41–50	4
51–60	4
61–70	14
71+	5
Tumour	
Lung	8
Colorectal	1
Ovarian	3
Breast	8
Renal	6
Malignant melanoma	5
Time since diagnosis^a^	
≤ 2 years	7
2–4 years	11
5–7 years	5
8–10 years	5
> 10 years	2*
IBP treatment^b^	
Monoclonal antibodies	4
Immunotherapies	1
CDK4/6 inhibitorsz	5
PARP inhibitors	3
Tyrosine kinase inhibitors	15
Combination	3

^a^ Time since diagnosis was not known for one participant.

^b^ Treatments for each category included: Monoclonal antibodies; pertuzumab, and cetuximab: Immunotherapies; avelumab, ipilimumab, pembrolizumab: CDK 4/6 inhibitors; abemacicilib, palbococlib: PARP inhibitors; niraparib: Tyrosine kinase inhibitors; crizotinib, palbococlib; loratinib, dafrafenib, soratinib, alectinib, palbocicilib, cabozantinib: Combination; ipilmumab and nivolumab, dabrafenib and trametinib, pertuzumab and trastuzumab. Where patients were utilising combinations of different classes of drugs, they have been categorised as “combination”.

**FIGURE 1:**
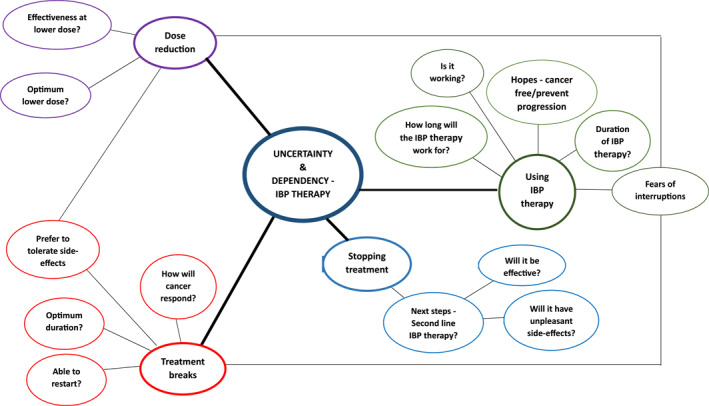
Dependency and uncertainty theme with sub‐themes and concepts.

Two overarching themes were selected of relevance to the research question, that is ‘what is the psychological impact of targeted therapies?’ These were (i) the psychological impact of a stage IV diagnosis and of living with advanced cancer; and (ii) uncertainty and dependency associated with IBP therapies (See Figure 1)

### 3.1 The Impact of the Diagnosis and of Living With Stage IV Cancer

Most participants' reaction to the diagnosis of, and living with, advanced cancer was shock and disbelief: the ‘*shock horror of the diagnosis was the pervading thing, the blanket of gloom it puts on your life and changes everything.’* (SU01) and the days following were described as ‘surreal’ or ‘dreamlike’. A few were relieved to receive the diagnosis particularly when it had taken time to discover the cause of their symptoms or were stoic and focussed solely on the next steps. Being given a diagnosis that suggested they were terminally ill yet there was treatment available, was difficult to comprehend for some. Illustrative quotations for the impact of a stage IV cancer diagnosis are shown in Table 2.

**TABLE 2:** Impact of a stage IV diagnosis.

Shock, disbelief and confusion
*I think, I was just in a dream, just didn't function, and I went on for a couple of days. SU09* *You've just been told you're dying. Then they talk about the future, and you think, well on the one hand, you tell me I'm dead, the next hand, you're telling me there's treatment, […]. And it is a bit confusing. We go into that day in great depth because it was a bad day for us. SU25*
Uncertainty over life‐expectancy
*When I first received (the diagnosis), I was pretty shocked, thought the world, the ground was just going to open and swallow us up, can't believe it's you, and just don't know what was going to happen to my family. SU06* *It was only when friends came round, when they found out and heard that about me because they would say, “how are you doing?” “I'm fine. I'm fine.” but all these things are going through your mind, just what you're going to do, what's going to happen? SU29*

Living with advanced cancer took its toll on the mental health of several participants: *‘You have your very dark times when you do wonder whether it's all worth carrying on*.’ (SU25). This was attributed to the treatment side‐effects some experienced and to the uncertainty of disease progression. Several thought about their cancer daily, their life expectancy and whether the cancer would return or progress. Although surviving beyond the expected timescale was a cause for celebration, they were always aware of living on borrowed time. Others had buried these thoughts and tried to make the most of life, taking each day as it comes, despite the constant reminders of medication and scans, pain if they neglected to take analgesics and exposure to fund‐raising cancer campaigns.

Participants had experienced changes to their sense of self through living with cancer or ‘*the normal you is blurred with the current you*’, (SU17). This included being more forthright whereas previously they were less outspoken, no longer being worried or angry about the minor things in life, or less able to deal with minor issues than previously. Having to give up work or leisure activities had impacted negatively on their sense of self and purpose. Illustrative quotes for the impact of living with stage IV cancer are shown in Table 3.

**TABLE 3:** The impact of living with stage IV cancer.

Mental state
*You have your very dark times when you do wonder whether it's all worth carrying on. SU25* *It started to get to the point where I suddenly lost any joy in anything. Nothing was enjoyable. You'd go somewhere but I found it hard to enjoy it, or it just seemed very flat and I couldn't be bothered with things. SU 17*
Make the most of life
*I just put it to the back of my head and say, well, it's not going to happen yet. Let's get on and do these dishes or make a sandwich or make a cup of tea or whatever I'm doing … Which is really helpful, I think. If I was a worrier, it could affect my mental health, I'm sure. SU18* *I go with whatever the medical staff at the hospitals tell you should be doing, giving you medication, you just focus on that, and take every day at a time. Try not to think too much into the future, And yeah, I feel .. I've got to somewhere I feel okay. I get a bit scared every time I have to have a scan. But I'm presuming that's quite a normal reaction.’ SU14*
Will cancer return or progress?
*I'm living with cancer because they can't say whether I'm free or not. […] recently, after my last scan, I asked how long I would be on immunotherapy […] and he said, “Well, we could talk about you coming off it now but I can't say that you would not get it again.” … But I can't say I'm ever going to be free now. […] I'm hoping that I will have cracked it this time, but on previous history, he can't say it won't come back. SU31* *They think (a lump) probably is just scar tissue, but nobody can give you a definitive answer. That really messes with you because you think, “Well what is it?” poke, poke, poke. Has it got bigger? Yes, it's got bigger. SU10*
Living on borrowed time?
*It's when it comes up to the date of when I was told, that's when I think about it and now, I've got this little devil on this side and a little angel on this side and the devil says, “You're coming up to 10 years. You're not going to last any longer,” and then this one goes, “Yes, but I was told 2 months and I'm still here now”. SU29* *You start thinking well if it's been stable so long, and we know it's not going to carry on forever, are we getting closer, are we getting further away from the bad day? So, no, it doesn't, it might lessen, but it certainly doesn't go away, the thought death is around the corner. SU25*
Changes to sense of self
*You try and be what you want, not what everybody else wants, it's being a bit more selfish*. SU29 *Somehow I seem to have got through this big thing but throw me a small thing and I'm floored.’*SU28 *I used to go skiing every year and all that disappeared. So you start to feel like an old man, rather than whatever you felt like beforehand*. SU24

### 3.2 Dependency and Uncertainty Associated With IBP Therapies. *‘It's Only Because of These Tablets I've Lasted This Long’*

Fear of cancer recurrence or progression were associated with the effectiveness of treatment, treatment breaks and changing therapies. Feelings of uncertainly and dependency were described for people using IBP therapy along the treatment pathway, as summarised below (quotations in Table 4).

**TABLE 4:** Additional quotations for uncertainty and dependency in relation to IBP therapies.

Using IBP therapy
*Hopes are that it continues to work and shrinks the tumour and stops any spread in which case I'm going to have a few more years […] I'd like to say a year or two, that would be great. SU08* *Not knowing whether it's working, waiting for scan results is the worst thing. It's 2 weeks going into you know you've got a scan booked and then waiting for the results afterwards is an absolute nightmare. I had a scan 3 weeks ago now and my next appointment is not for another 3 weeks. SU10* *Luckily, it is working. Within the first month of taking it, my first scan came back and I was clear, and every scan since then has been clear of no cancer. Obviously, I am still taking it, so I'm not in remission. I don't know… if I'm going to be on these forever or for life, or if I am going to be, hopefully, getting to remission, but they're just taking it year by year now because of how evasive it was and how quickly it spread. SU04* *With treatment, because there's so many unknowns about it … there's lots of different viewpoints from different consultants as well and then knowing as well how it's going to work, how it's going to affect really does mess with your head because you just don't know what's going to happen. SU10*
Treatment breaks
*I'm too fearful to have any break from it, even though I'm classed as stable, you know, no changes. But I just think, if you had a break and then things changed, you'd never know whether that was going to happen anyway, or whether it's the break that's made it happen. SU17* *It used to be very worrying because you'd set off on a snowy morning thinking […], “God, I hope I get there.” You're not thinking, “I hope I get there because it's snowing.” You're thinking, “I hope I can get there because I've got a load of drugs that my life depends on. If I haven't got them then what do I do?” It's really not nice. SU02*
Dose reduction
*I think I would be anxious that the recommended dose is what I take so if I'm not taking that recommended dose then is it going to have the same effect that the recommended dose would have? SU12* *I'm on the highest dose, so they did say if it did get too much, I could either take a break or they could reduce it. But I was like, “No, I'm going to power through this”. SU04*
Stopping treatment
*When they said the first lot didn't work, they stopped it for a little while until they could do scans and see other doctors and talk to other oncologists about the best way to go. It was a month or so that I didn't have any treatment. It was like being in limbo and I was just hanging thinking, “What's happening?” SU13* *I don't know whether it's great if it's a second line, and it's great if it's a third line. And … are they going to be as longstanding as the one that you're currently on or is it just like a temporary, short–term measure? […] the research will say, “Someone having this drug from someone who didn't have this drug went from 35 months to 40 months” And you think, “That's shit, isn't it, really?” I know it's an improvement but, you know, in reality what is that improvement? SU17*

#### 3.2.1 Using IBP Therapy

Hopes for the treatment varied between participants. Some, who through the treatment were cancer‐free, hoped it would prevent a recurrence. Others hoped that the therapy would rid them of cancer, or that it would stop any progression and ‘*keep it at bay’*,

Most participants were using relatively new therapies. As might be expected, and considering their diagnoses of advanced cancer, participants were anxious to know whether it was working.

With treatment, because there’s so many unknowns about it … there’s lots of different viewpoints from different consultants as well and then knowing as well how it’s going to work, how it’s going to affect really does mess with your head because you just don’t know what’s going to happen.

SU10

The clinical teams measured the effectiveness of the treatment through scans. Waiting for the scan result was described as ‘*the worst thing’*, because of the time to obtain the results. Over time, and with subsequent evidence from scans, participants were aware that the therapy had proven to be effective. Along with the delight expressed by participants that it had shrunk the cancer and/or prevented it from progressing was the awareness there would come a time when the treatment would cease to work. There was uncertainty as to how long it would continue to be effective. One participant had read about the experiences of others from patient websites and found there were those ‘*who've been alive for a long time on it’* and those who had deteriorated after the treatment stopped working (SU24). Questions were raised as to whether, following successive scans that demonstrated they no longer had cancer, they would continue treatment. This was particularly important for those who were experiencing treatment side‐effects.

Some participants considered their IBP therapy as saving and prolonging their lives: *‘It was like a breath of fresh air. I'm not going to die yet’.* (SU18). This was especially so for participants who had survived well beyond the time predicted:

It's only because of these tablets, I've lasted this long because in 2014, I asked what the prognosis was, and (consultant) said, 16 months. So to be here, seven years later, is fantastic. I can only say it's those tablets that have kept me going so long

SU03

IBP therapies were described as ‘marvellous’, ‘fantastic’ and ‘amazing’ and there were numerous accounts about their effectiveness. Participants shared how delighted their clinical team were with the outcome of the therapy. In several cases, the IBP therapy had been more successful than initially envisaged: ‘*I was a statistic that (*consultant*) was taking to conferences’*, (SU01).

I could see the oncologist was really happy with how much it had shrunk the tumour … And the reaction from the staff to the results of the scans was like kind of, oh, this is really good. I'm glad to put you on that medication.

SU14

In acknowledgement of the perceived effectiveness, participants described becoming dependent on, and having uncertainties about, their IBP therapy. These were linked to fears and anxiety about any interruption to the treatment regimen including changes to the therapy and its effectiveness in preventing the cancer from progressing.

The more important bit is at times when it seemed (targeted therapy) wasn’t working as well … it was then that I was realising that I’m psychologically dependent on that drug. It’s my prop. The idea of being without it is frightening.

SU01

#### 3.2.2 Treatment Breaks

In the UK, clinicians can offer patients unplanned breaks from IBP therapy, for example, if they are experiencing side effects from the treatment or require an intervention for an unrelated illness or medical emergency [18]. For most, the thought of taking a treatment break caused concerns. Firstly, participants described about uncertainty how the cancer would respond if treatment was stopped even for a short period.

The last break was four weeks off medication, and I get worried when I’m on no medication for that length of time. I think: “What’s happening in your body when you’re not on that medication for a whole four weeks?” So that tends to play on your mind a bit.

SU05

Secondly, there was uncertainty around the optimum treatment break duration. Participants talked of the therapeutic value of the treatment and questioned why it is acceptable to stop it. One mentioned the need for proof that the cancer will not progress during a treatment break.

If you’re diagnosed with metastatic cancer and it’s treatable and you’re on treatment, you’re on it for a reason. You’re not on it because you can suspend it. I just don’t like this, “Well you’ll be alright for a couple of months.” It’s like, “Really? Well you prove that I’m alright for a few months”.

SU02

Thirdly, participants were concerned that if they stopped it may not be possible to restart it. One believed that treatment breaks may be an economic initiative driven by the high cost of targeted therapies.

They said potentially, if I come off it, I might not be able to go back on it. … I can’t remember what reason they gave us. But yes, they did say, I may not be able to come back on it or I might have to pay for it.

SU04

One participant had considered taking a treatment break because of unpleasant side effects and discussed at length with their partner that if they did stop treatment, they may be reluctant to resume it: ‘*if we were to do that, and I felt so much better, would I go back on the drug or not? And I couldn't answer the question. I don't know what I would do in that circumstance’*, (SU25).

Because of concerns about the cancer progressing several participants who were experiencing unpleasant side effects preferred to tolerate them rather than take a treatment break.

I would rather put up with a little bit of, the side–effects I have, I’d rather put up with that than face things going downhill.

SU17

#### 3.2.3 Dose Reduction

Similarly to the opinions about treatment breaks, some participants questioned why, when there is a recommended dose, they should not be taking that dose. There were concerns that the therapy would be less effective at a lower dose.

If I need this dosage to keep the cancer at bay, then fine give me the dosage.

SU16

Participants needed to be certain that a reduced dose was still effective in treating the cancer or preventing it from progressing. One participant said they were happy to reduce their dose ‘*as long it's still working*’ (SU05). Another said that without this evidence they would rather remain on the current dose.

I always think that there’s a reason why you’re on the higher dose. If they know that’s the therapeutic dose, I don’t want to reduce it without really, really strong evidence it doesn’t compromise your treatment. I would rather stay on it unless it was really, really unbearable.

SU02

Only a few participants reported they were aware that the IBP therapy was still effective at a lower dose: one had read this in the drug information leaflet and on the internet; the other was told by their clinician ‘*that reducing the dose doesn't necessarily mean it's not going to continue to be beneficial.’* (SU08).

Again, participants had a preference to endure the treatment side effects rather than have a dose reduction. This was linked to uncertainty around the optimum dose to manage their cancer.

I’m perfectly happy to stay on the dosage and be safe, rather than just come off it for the sake of diarrhoea. I can manage that.

SU16

#### 3.2.4 Stopping Treatment

*Several participants were concerned about the next line of treatment including potential side effects.* For a small number of participants, the treatment had not worked from first being prescribed or, after some time, had ceased to be effective. For the former, being without any treatment while the clinical team considered what to do next was a difficult time.

When a treatment, that had been much more effective and for longer than the clinical team had envisaged, stopped working this was particularly hard to accept.

I’d come to believe, because I wanted to, that I could be on crizotinib forever and nothing would change for the worse. That’s what I wanted to believe. The reality has been brought home to me that it has a limited shelf life, it’s just a much bigger shelf for me than it has been for a lot of people.

SU01

There was uncertainty over, and concerns about, what the next steps were if, and when, the therapy stopped working. In the event of this happening, participants talked of the importance of knowing the next steps in terms of other treatments. One participant felt they had lost so much control over their lives through cancer, and being informed of subsequent potential treatments would at least given them some control over decisions on what to try next. Although another participant had been informed by their clinical team that ‘*another therapy might be being invented that might become available’ (SU22)* they talked of their concerns of not knowing what will happen next:

Obviously, it’s always a bit, it’s kind of on your shoulder, of what happens if these drugs stop working, because I do know that one day they will, that the cancer will start fighting back and it will become used to the treatment and find a way around it … It is a worry and I think what will happen next […] what will I go onto, what treatment?’

SU22

Another issue related to the effectiveness of subsequent treatments and if they would work as well as current therapy. Participants had heard of second‐ and third‐line treatment but did not understand what that meant particularly in relation to effectiveness. Gains in life expectancy that may be encouraging to the research community were of little comfort to people with advanced cancer.

A few participants were aware of what the second line of treatment would be if their current therapy ceased to be effective, and there were concerns about the side effects. Others, who did not know, assumed the side effects would be unpleasant. This was based on the belief that the clinical team had begun with a therapy that had fewer side effects: ‘*This first line is probably the least severe’,* (SU01). Participants reflected on, and weighed up, a potential future life with more severe side effects from a different therapy.

To have a sense of not just how much time have I got, although that’s important, but also how much time have I got living a pretty normal life? The mabs (monoclonal antibodies) have minimal side effects but I know the second line of treatment has more side effects, probably the next one for me would be something called Kadcyla and everyone says it makes the skin on the palms of your hands and the soles of your feet fall off which is quite horrible.

SU19

## 4 Discussion

This study is the first to examine the psychological impacts of IBP therapies from the perspective of individuals with advanced cancer. Although participants' reactions to the diagnosis of, and living with, advanced cancer resonated with previous research [12, 13, 14, 15, 16] the study has several novel findings. First, participants experienced uncertainty at various stages of IBP treatment, including using the therapy, treatment breaks, dose adjustments, and potential treatment cessation. Second, individuals reported forming an attachment to their IBP treatment, linking the use to positive cancer outcomes. Third, participants expressed anxiety or concern about the possibility of not receiving their therapy, both in the short‐term (e.g., missing an appointment due to weather) and long‐term (e.g., discontinuing treatment due to side effects), often describing these feelings as a sense of “dependence”.

**FIGURE 2:**
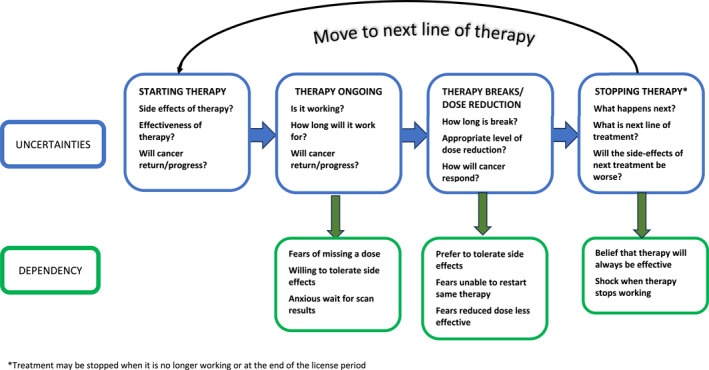
IBP treatment cycle, uncertainties and dependency factors. *Treatment may be stopped when it is no longer working or at the end of the license period.

The term “dependence”, as described by study participants, has been defined in the literature as ‘the state of relying on or needing someone or something for aid, support, or the like’ [19]. There are two types of drug dependence: (i) physical dependence, and (ii) psychological dependence. Physical dependence relates to the adaptation of a physiological process in the presence of a drug, while psychological dependence involves the emotional or mental attachment to a drug [20]. The findings from this work suggest that people using IBP therapies develop psychological dependence towards these treatments. Although psychological dependence has been reported for certain types of drugs (e.g., stimulants [21]), to our knowledge, this has not been reported for the IBP therapies. Indeed, stopping a medication—or proposing to stop a medication—when a person has psychological dependence can result in the development of withdrawal symptoms, such as anxiety. Feelings of nervousness and anxiety, as well as uncertainty, were reported amongst our participants, particularly when a treatment break, dose reduction or cessation of therapy was being considered. Figure 2 illustrates the IBP treatment cycle, showing the points of uncertainty and possible dependency. If a patient moves to a second‐ or third‐line IBP treatment, they restart the cycle and, again, can experience uncertainty and dependency with new IBP treatment.

Although this is the first study exploring the psychological impacts of IBP therapy, previous work has reported psychological distress for people receiving more traditional cancer treatments, including radiotherapy [22], and surgery [23, 24]. In contrast to this study, feelings of uncertainty and dependency have not been described in people receiving more traditional cancer treatments. Research exploring the experiences using IBP therapies has, however, started to emerge, although this work has often focussed on specific aspects of treatment. For example, Zwanenburg and colleagues aimed to explore the lived experiences of long‐term responders to immunotherapy or targeted therapy [25]. The work showed that people perceived themselves to be living in the “twilight zone”, where they did not feel “healthy” nor like a “cancer patient”. It was also reported that these feelings led to challenges around perceptions related to identity, uncertainty and adapting to the new normal. Our work builds on the literature and shows that the feelings of uncertainty do not exclusively relate to long‐term responders of IBP treatment, but rather this uncertainty can be experienced from the start of IBP therapy and across the treatment journey. To our knowledge, no other studies have reported people experiencing psychological dependency towards their cancer treatment—whether this is with the more traditional treatments, such as radiotherapy, or the newer IBP therapies.

### 4.1 Clinical Implications

The practice and policy implications of this work are important: specifically, our findings suggest that when people are prescribed IBP therapies, there should be consideration of providing additional tailored psychological support to deal with uncertainty associated with their use, and possible psychological dependency. This support should be broader than the traditional monitoring and management of treatment adverse effects. Specifically, it should be targeted at different points in the IBP treatment cycle where uncertainly and dependency are likely to occur (Figure 2). For example, when initiating these therapies, it is important there is appropriate support and education in place to ensure the patient does not have incorrect or misguided treatment beliefs. Furthermore, there should be appropriate support in place to cope with the uncertainty and possible psychological dependence when there is consideration of a treatment break, a dose reduction or possible cessation of therapy. For some IBP therapies, these support points could be proactively planned according to the treatment schedule; for example, in some healthcare systems, immunotherapies are used for a defined time‐period and then stopped (in the UK, informed by findings from the KEYNOTE024 trial [26], pembrolizumab, for PD‐L1‐positive metastatic non‐small cell lung cancer, is given for a maximum of 24 months and then stopped [27]). There is extensive literature examining the effectiveness of psychotherapeutic interventions [28, 29, 30, 31] and, in the past few decades, there has been focus on screening people with cancer for distress, anxiety, depression, and demoralisation [32]. It is acknowledged that oncology nurses are well placed to provide initial screening assessments for people starting cancer treatment and, when needed, referrals can be made to more specialist psycho‐oncology professionals [33]. Future research could explore the best supportive psychotherapeutic strategies to use for people using IBP therapies at the different treatment points, and whether oncology nurses are happy to take on this responsibility and, importantly, are competent to do so.

### 4.2 Study Limitations

Our study included a diverse sample of people with different cancers using a range of different IBP therapies. This is a key strength of the work. However, we did not formally record how long participants had been on IBP therapies. The interviews were undertaken at one point in the treatment journey, and some participants may have used IBP therapy for only several months and not discussed treatment breaks or dose reductions, while others may have been using IBP therapies for several years and, accordingly, had these discussions. It is possible that the feelings of uncertainty and psychological dependency reported by participants vary over time, according to the stage of the treatment journey. For example, as someone becomes more familiar with their IBP treatment, does the uncertainty associated with it diminish and the psychological dependency intensify? Our study did not account for this. Future work could seek to follow people longitudinally to explore whether feelings of uncertainty and psychological dependency change over time. Future work could also explore the role of caregivers and family members in supporting the psychological needs of people using IBP therapies.

We had low numbers of participants for some cancer types which rendered a formal comparison difficult. However, informal analysis did not suggest that dependency and uncertainty were more prevalent in one specific cancer group or time on IBP therapies. These were issues which appeared to be independent of cancer type and therapy duration.

## 5 Conclusions

Alongside the growing availability of IBP therapies, it is important that patients' support needs are met. Here, feelings of uncertainty and psychological dependency towards IBP therapies were described. People using IBP therapies may require additional tailored psychological support throughout treatment that is broader than monitoring and managing IBP treatment‐related adverse effects.

## Funding

This work is funded by a project grant from Macmillan Cancer Support, award number: FO‐7165351.
